# Supplementary material for: The causal role of circulating inflammatory markers in osteoporosis: a bidirectional Mendelian randomized study
Source: Front Immunol. 2024 Jul 18;15:1412298. doi: 10.3389/fimmu.2024.1412298 (PMC11291241; doi:10.3389/fimmu.2024.1412298)
Supplement: Supplementary file 1 [file DataSheet_1.zip › supplementary material/CIM-OSTEOPOROSIS /GCST90274805.csv_scatter.pdf]

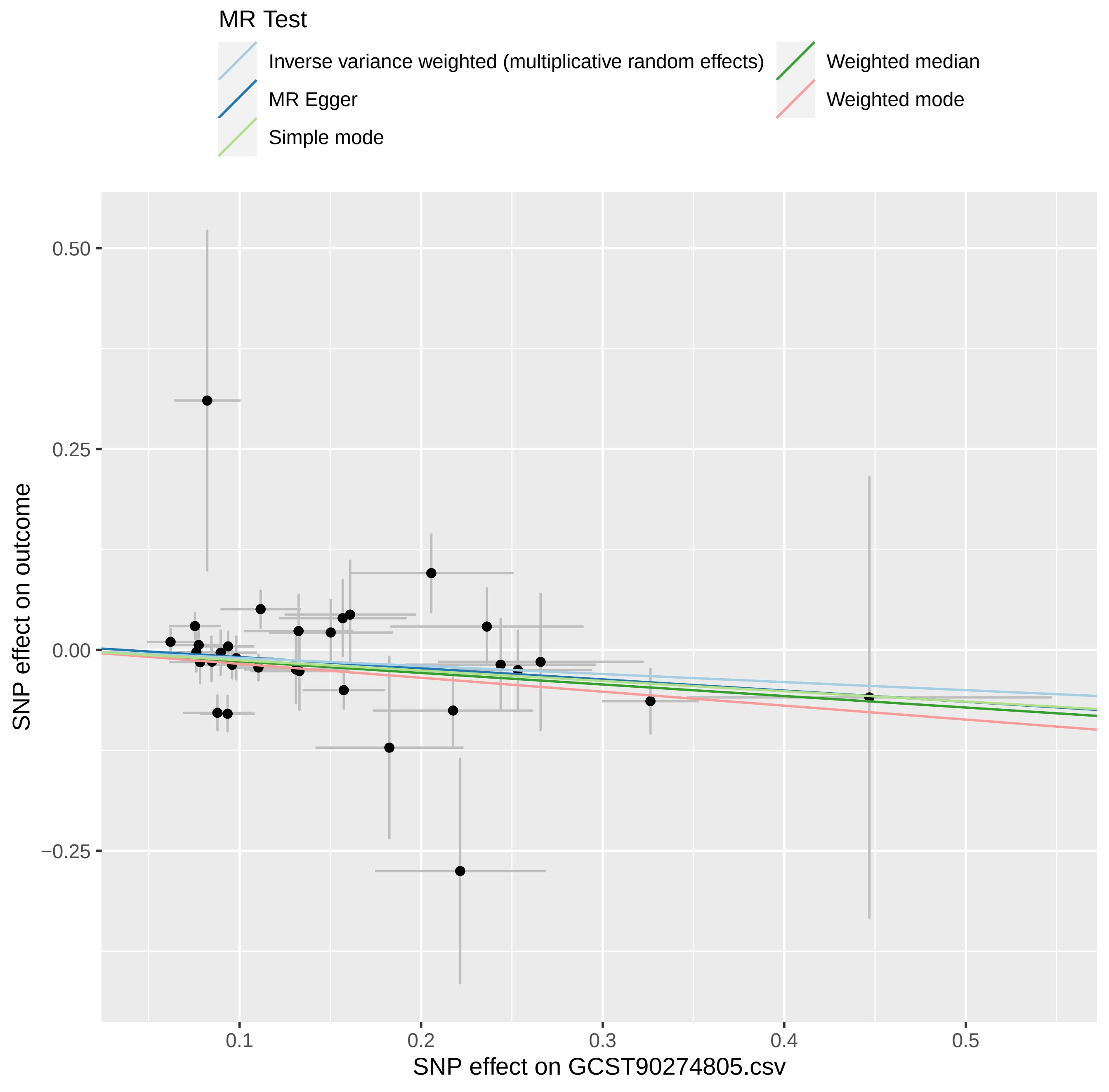

MR Test
Inverse variance weighted (multiplicative random effects)
MR Egger
Simple mode
Weighted median
Weighted mode
0.50
0.25
0.00
−0.25
SNP effect on outcome
0.1
0.2
0.3
0.4
0.5
SNP effect on GCST90274805.csv
